# Properties of Polysiloxane/Nanosilica Nanodielectrics for Wearable Electronic Devices

**DOI:** 10.3390/nano12010095

**Published:** 2021-12-29

**Authors:** Elena Ruxandra Radu, Denis Mihaela Panaitescu, Laura Andrei, Florin Ciuprina, Cristian Andi Nicolae, Augusta Raluca Gabor, Roxana Truşcă

**Affiliations:** 1 National Institute for R&D in Chemistry and Petrochemistry ICECHIM, 202 Spl. Indendentei, 060021 Bucharest, Romania; radu.elena.ruxandra@gmail.com (E.R.R.); cristian.nicolae@icechim.ro (C.A.N.); raluca.gabor@icechim.ro (A.R.G.); 2 ELMAT Laboratory, Faculty of Electrical Engineering, University Politehnica of Bucharest, 313 Spl. Indendentei, 060042 Bucharest, Romania; laura@elmat.pub.ro; 3 National Research Centre for Micro and Nanomaterials, University Politehnica of Bucharest, 313 Spl. Indendentei, 060042 Bucharest, Romania; truscaroxana@yahoo.com

**Keywords:** nanocomposites, dielectric properties, DMA, flexible electronics, polysiloxanes, silica nanoparticles

## Abstract

Polymer nanodielectrics characterized by good flexibility, processability, low dielectric loss and high dielectric permittivity are materials of interest for wearable electronic devices and intelligent textiles, and are highly in demand in robotics. In this study, an easily scalable and environmentally friendly method was applied to obtain polysiloxane/nanosilica nanocomposites with a large content of nanofiller, of up to 30% by weight. Nanosilica was dispersed both as individual particles and as agglomerates; in nanocomposites with a lower amount of filler, the former prevailed, and at over 20 wt% nanosilica the agglomerates predominated. An improvement of both the tensile strength and modulus was observed for nanocomposites with 5–15 wt% nanosilica, and a strong increase of the storage modulus was observed with the increase of nanofiller concentration. Furthermore, an increase of the storage modulus of up to seven times was observed in the nanocomposites with 30 wt% nanosilica. The tensile modulus was well fitted by models that consider the aggregation of nanoparticles and the role of the interface. The dielectric spectra showed an increase of the real part of the complex relative permittivity with 33% for 30 wt% nanosilica in nanocomposites at a frequency of 1 KHz, whereas the loss tangent values were lower than 0.02 for all tested nanodielectrics in the radio frequency range between 1 KHz and 1 MHz. The polysiloxane–nanosilica nanocomposites developed in this work showed good flexibility; however, they also showed increased stiffness along with a stronger dielectric response than the unfilled polysiloxane, which recommends them as dielectric substrates for wearable electronic devices.

## 1. Introduction

Flexible electronics have emerged as a distinct field of research due to the increased need for soft, flexible or stretchable electronic systems for biomedical applications, robotics, intelligent textiles and wearable electronic devices, among others [1,2]. Polymers are the preferred materials for flexible electronic devices due to their low stiffness and amendable dielectric properties [1]. Great progress has been made in the design of biointegrated electronics, shape conformable electrodes or electro-active nanomaterials [1,2,3,4]. Although attractive, due to easy manufacturing of objects with various shapes and size, low cost, chemical resistance, high breakdown strength and low dielectric loss, most polymers are characterized by a low dielectric constant and therefore a low energy density. For large application in modern electronic devices, the energy density of polymer dielectrics should be improved. The most facile route to increase the energy density in polymers is the incorporation of nanofillers characterized by a high polarization and the manufacture of dielectric nanocomposites [5]. Thus, polymer nanodielectrics with high dielectric permittivity and good breakdown strength are promising for use in energy storage and energy conversion devices [6,7]. As they are attractive characteristics for use in these applications, the benefits of the polymer matrix, such as its lightweight properties, low-cost and easy processing, should be maintained in nanodielectrics.

However, increasing the relative permittivity while maintaining a low dielectric loss in polymer nanodielectrics is a challenging task [3,5]. Some progress has been made in this direction by designing polyvinylidene fluoride (PVdF) nanocomposites with several fillers, especially with BaTiO_3_ [8]. It was reported that core–shell PVdF–BaTiO_3_ nanoparticles increased the dielectric constant of PVdF from less than 10 to almost 30 at 1 kHz. However, ferroelectric polymers, such as PVdF, are not soft and they may cause large polarization hysteresis and high dielectric loss under high electric fields [9]. This may lead to premature failure of the devices. Therefore, nonferroelectric polymers have gained increased interest, provided that their dielectric and mechanical properties are improved.

Some of the most used materials for flexible dielectrics are polysiloxanes, such as poly(dimethylsiloxane) (PDMS), which are characterized by a fully saturated backbone of alternating silicon and oxygen atoms. This structure results in excellent properties, such as a high elasticity even at low-temperature, high-temperature resistance, resistance to radiation, chemicals and climatic constraints, easy processability and biocompatibility [10,11,12,13,14,15]. However, the mechanical properties, and especially the stiffness, of unfilled polysiloxanes are poor. In addition, as most polymers, polysiloxanes are characterized by a low dielectric permittivity [16,17,18,19,20], which is an advantage for some applications, such as insulators, but not for energy storage and energy conversion devices. Either dielectric fillers, such as barium titanate, lead magnesium niobate, lead titanate, zinc oxide and titanium dioxide, or conducting fillers, such as carbon black, carbon nanotubes and graphene oxide, have been tested to increase the dielectric permittivity of polysiloxanes [15,17,18,19]. In these studies, the improvement of the dielectric properties was largely accompanied by the modification of other properties.

Nanosilica has commonly been used to modify the mechanical properties of polysiloxanes due to its good price–performance ratio [13,14]. The size of the silica particles has a strong influence on the mechanical properties of polysiloxane composites; in one study, a monotonic increase of the tensile and tear strengths along with fracture toughness of polysiloxane composites was observed when the size of silica particles decreased from 500 to 20 nm [2]. The nanosilica can bond to the polysiloxane chains through physical (van der Waals and hydrogen bonding) or chemical bonds, resulting a complex network [2,13,21]. Although polysiloxanes have a very low surface energy and weak adhesion properties, the interfacial interactions between PDMS and silica may determine a better effect of this filler compared to other inorganic particles. Thus, compared to aluminum hydroxide of similar size, the influence of silica particles on the mechanical properties of silicon rubber has been shown to be better [22].

Nevertheless, previous works have shown the great difficulty of homogenously incorporating nanosilica in polysiloxanes to take full advantage of the polymer matrix and nanofiller properties [20,21,22,23]. Despite their chemical similarity, a good dispersion of nanosilica in polysiloxanes may be challenging. Pioneering work has shown a broad distribution of particle sizes in silica-reinforced silicone rubber and the presence of large aggregates, 20–30 µm in size [23]. Several routes were tested to improve the dispersion of micro- and nanosilica particles in polysiloxanes, such as: (i) mechanical blending of nanosilica with PDMS suspension followed by crosslinking [24]; (ii) in situ sol–gel process for the generation of silica particles in the polysiloxane [25,26]; (iii) ultrasonication and miniemulsion polymerization with core–shell silica nanoparticles encapsulated by PDMS [27]; (iv) surface modification of nanosilica by grafting silane coupling agents [2,13] or other modifiers, such as fatty alcohols [28], and then blending with the silicon rubber; (v) addition of a silicone oil to decrease filler–filler interactions and irradiation crosslinking [29]; (vi) anionic ring-opening polymerization of dimethylcyclosiloxane and nanosilica addition followed by crosslinking [21]; or (vii) addition of an adhesion promoter and surface modified silica nanoparticles in the silicone rubber for improving compatibility and mechanical properties [30]. However, all these methods were not able to completely eliminate the large nanoparticle aggregates [24,25,26] or control the crosslink density and the size of silica microdomains [25,26]. Moreover, the occurrence of rather complicated intermediate operations in the manufacturing process makes the control of the properties more difficult [27,30]. In addition, the use of organic solvents to allow the incorporation of a large amount of silica into polysiloxanes [15,20,24] raises serious environmental issues. Besides, all these works studied only the improvement of the mechanical properties of polysiloxanes, and the issue of their poor mechanical and dielectric properties has not been addressed so far in a unitary way. This goal is crucial because even a slight increase in permittivity leads to a reduction in the size of portable devices, which is extremely important in practice.

In this study, an easily scalable at industrial level and environmentally friendly method was applied to obtain polysiloxane/nanosilica nanocomposites with a large content of 20 nm nanosilica. The nanofiller was intensively mixed with the silicone part containing the catalyst, and then with the part containing the crosslinker in a mixing chamber equipped with two sigma rotors, counter-rotating towards each other. The influence of high amounts of nanosilica on both the mechanical and dielectric properties of polysiloxane was studied in this work. The simultaneous improvement of stiffness and dielectric permittivity in the studied polysiloxane/nanosilica nanodielectrics is promising for the application of these materials for flexible electronic devices.

## 2. Materials and Methods

### 2.1. Materials

A commercial polysiloxane, Elastosil LR 3003/40, was provided by Wacker Chemie (Munich, Germany) and nanosilica with an average particle diameter of 20 nm, density 2.2 g/cm^3^ and bulk density 0.011 g/cm^3^ was supplied by Sigma-Aldrich Co. (Saint Louis, MO, USA).

### 2.2. Preparation of Nanocomposites

Equal parts of Elastosil LR 3003/40 A and B along with different amounts of nanosilica were mixed at room temperature for 10 min with 100 min^−^^1^ in the mixing chamber of a Brabender LabStation (Duisburg, Germany) equipped with two counter-rotating sigma rotors. Firstly, the Elastosil part A, which contained the catalyst, was intensively mixed with the nanosilica and then with the part B, containing the crosslinker. After homogenization, the mixtures were thermally crosslinked into a Dr. Collin Press (Ebersberg, Germany) using 150 × 150 × 0.7 mm metal frames and the following conditions: 165 °C, 5 MPa, 5 min. The pressed sheets were placed in an oven at 200 °C for 6 h for post-crosslinking. The polysiloxane/nanosilica nanocomposites with 0, 5, 10, 15, 20, 25 and 30 wt% nanosilica were denoted “Ex”, where “x” is the nanosilica concentration (wt%).

### 2.3. Morphological Characterization

The morphology of the polysiloxane/nanosilica nanocomposites was investigated by scanning electron microscopy (SEM) using a Quanta Inspect F Scanning Electron Microscope (FEI-Philips, Hillsboro, OR, USA) with a resolution of 1.2 nm. The morphology was observed on the surface of the specimens and in sections obtained by cryo-fracturing in liquid nitrogen, after sputter-coating with gold for better contrast. The samples were investigated with an accelerating voltage of 30 kV at the surface and with 10 kV in section.

### 2.4. Thermal Properties

The thermogravimetric analysis (TGA) was carried out on duplicate samples of nanocomposites of 16–20 mg with TA-Q5000 equipment (TA Instruments Inc., New Castle, DE, USA). The thermal behavior was analyzed from room temperature to 1000 °C with 10°/min under nitrogen flow (40 mL/min).

The melting/crystallization events were analyzed by differential scanning calorimetry (DSC) under helium flow (25 mL/min) using Q2000 V24.9 equipment from TA Instruments Inc. (New Castle, DE, USA). Approximately 15 mg from each sample were quenched to −90 °C and, after temperature equilibration for 10 min, heated to 55 °C, cooled to −90 °C and heated again to 55 °C. The samples were heated/cooled at a rate of 10 °C/min and maintained at −90 and 55 °C for 5 min for equilibration before recording data. The crystallinity (X_c_) of nanocomposites was calculated according to [11].

### 2.5. Mechanical Properties

Dynamic mechanical analysis (DMA) was carried out using DMA Q800 equipment (TA Instruments Inc., New Castle, DE, USA) in multifrequency-strain mode with a tension clamp. Specimens with the dimensions 12.7 mm × 6.5 mm × 0.7 mm (length × width × thickness) were rapidly cooled to −60 °C, where they were maintained for 1 min, then heated to 40 °C with a heating rate of 3 °C/min.

The tensile properties of the nanocomposites were determined at room temperature according to ISO 37 with an Instron universal testing machine model 3382 (Instron, Norwood, MA, USA) with a 2 kN cell. Five specimens were tested from each sample using a crosshead speed of 200 mm/min. Mean values and standard deviations of tensile strength and modulus were automatically calculated with Bluehill software.

### 2.6. Contact Angle

The surface hydrophobicity was evaluated at room temperature and ambient humidity using a CAM 200 from Biolin Scientific (Gothenburg, Sweden). Twenty-five drops of deionized water were dispensed on the surface of each sample with dimensions of 50 mm × 10 mm × 0.7 mm with an auto-dispenser and visualized with a high-resolution camera (Basler A602f). The contact angle was calculated for each drop by the CAM software using the Young equation. The results are shown as the mean of the twenty-five measurements.

### 2.7. Dielectric Characterization

Dielectric properties were obtained via dielectric spectroscopy using a Novocontrol Alpha-A Analyzer (Montabaur, Germany) with Active Sample Cell ZGS. The real part of the complex relative permittivity (ε_r_’) and the loss tangent (tan δ) were determined over a frequency range from 10^−2^ Hz to 10^6^ Hz by increasing the temperature from 30 to 80 °C with a step of 10 °C. Before measurement, the samples were kept for 10 min at each temperature under air flow for equilibration.

## 3. Results

### 3.1. SEM Investigation

**Figure 1:**
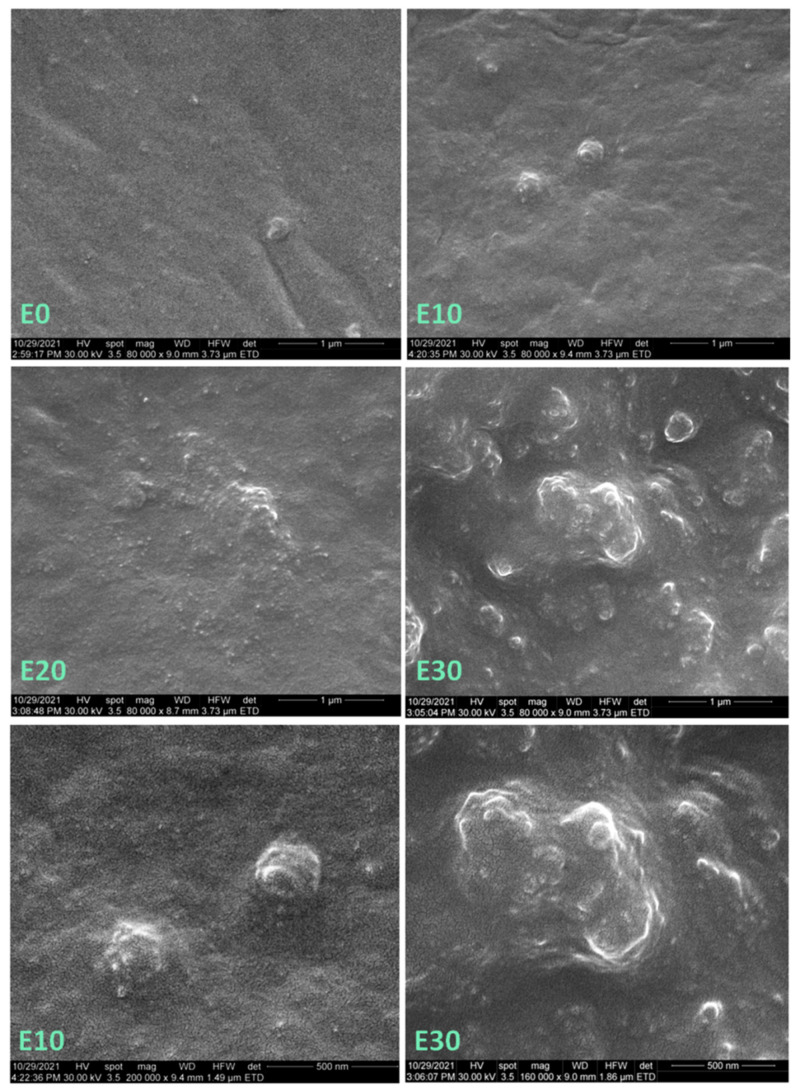
SEM images on the surface of E0, E10, E20, and E30 at ×80,000 magnification (top and center); higher magnification SEM images of E10 (×200,000) and E30 (×160,000) (bottom).

SEM images of the surfaces of polysiloxane and the nanocomposites, with different magnifications, are shown in Figure 1. The SEM image of E0 (unmodified polysiloxane) shows micro and nanoparticles with sizes from 100 nm to 1 µm. Therefore, the commercial polysiloxane contains a filler. In general, polysiloxanes are filled with small amounts of nanosilica which can be observed as individual nanoparticles or as clusters [31]. Many individual nanoparticles of 15–25 nm and a few larger particles (clusters) of about 200 nm in size were seen on the surface of E10, containing 10 wt% nanosilica. The SEM image with higher magnification of these clusters shows that they are composed of more than 20 nanoparticles of 20 nm, tightly surrounded by the polymer (Figure 1, E10). A similar morphology consisting of individual nanoparticles of 15–25 nm and agglomerations of 300–400 nm was observed in the case of E20 (Figure 1, E20). However, the nanoparticle agglomerations were larger and more numerous (Figure 1).

More such agglomerations on extended areas were observed on the surface of E30 (Figure 1) due to the increased concentration of nanosilica, which can be dispersed homogeneously with great difficulty. A large agglomeration of nanoparticles impregnated with polymer, which has a size of about 1 µm, can be observed in the detailed SEM image of E30 (Figure 1). In general, at this concentration, both individual and agglomerated nanosilica particles are very close to each other at distances similar to their size.

**Figure 2:**
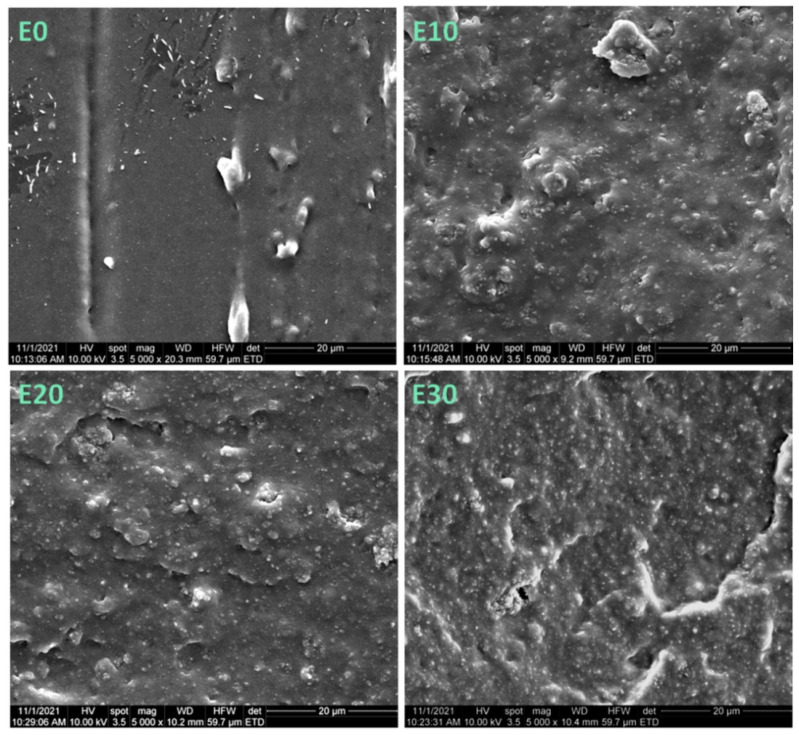
SEM images of the cryo-fractured sections of E0, E10, E20 and E30 nanocomposites.

**Figure 3:**
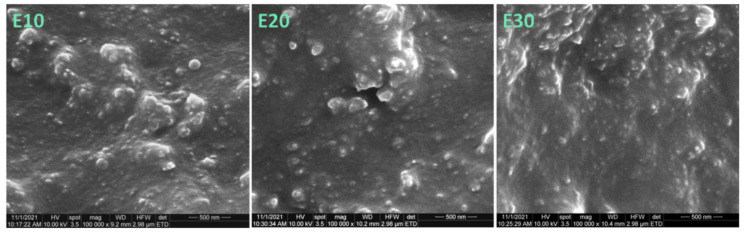
SEM images (×100,000) of cryo-fractured sections of E10, E20 and E30 nanocomposites.

The SEM images of fractured samples (Figure 2 and Figure 3) show new morphological details in the section of neat polysiloxane and the nanocomposites. Individual nanoparticles and clusters were observed in all the samples; however, the section of E0 was smoother than that of the nanocomposites, which were very rough. The roughness was explained by the formation of a network of nanosilica and molecular chains of the polymer, which may increase the mechanical properties of nanocomposites [30]. It should be remarked that no cracks and voids were seen in the sections of the nanocomposites, showing good adhesion between the nanosilica particles and the polysiloxane matrix.

### 3.2. Mechanical Properties

#### 3.2.1. DMA Results

**Figure 4:**
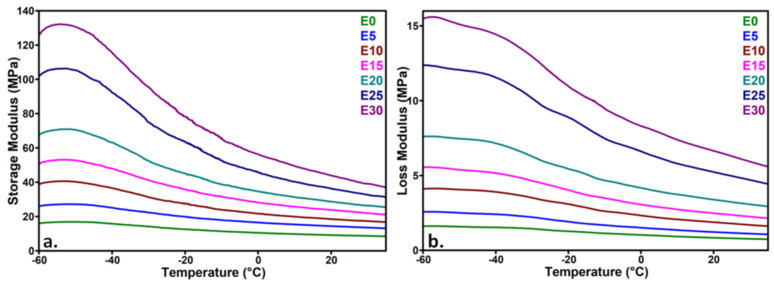
DMA curves of nanocomposites: (**a**) storage modulus and (**b**) loss modulus vs. temperature.

Figure 4 shows the variation of the storage modulus (E’) and loss modulus (E”) of the nanocomposites with temperature. A continuous decrease of E’ and E” with an increase of temperature was noticed due to the increased mobility of the polymer chains. The addition of nanosilica led to a strong increase of E’ and E” in nanocomposites. The increase of the storage modulus is related to higher energy storage, and the increase of the loss modulus is due to the viscous response and mechanical losses. As observed in Figure 4, the increase of the storage and loss moduli is not proportional with the concentration of nanosilica, with a higher increase observed for the nanocomposites with 25 and 30 wt% nanoparticles (Table 1). An increase of the storage modulus by seven times in the case of E30 and by five times for E25 was observed. At these concentrations, the formation of a network of nanoparticles in the polysiloxane matrix may be assumed, determining a leap in stiffness and viscous flow. Therefore, the interaction between nanoparticles and that between the polymer and nanoparticles will be enhanced in these nanocomposites and will restrict the mobility of polymer chains, influencing the mechanical properties. The agglomerations, observed at a high amount of nanosilica in the SEM images, will also contribute to the increased rigidity of the material.

A slight increase in the tan δ value with the concentration of nanosilica, regardless of the temperature, was observed in all the nanocomposites, except for E5 which contained the smallest amount of nanosilica (Table 1). A similar increase was reported for PDMS/silica (2–10%) nanocomposites obtained by a one-pot process consisting in simultaneous polymerization and silica generation. [32]. An increase in the tan δ value measured at the glass transition temperature is generally associated with an increased flexibility or weaker polymer–filler interactions [20]. However, the temperature range of DMA measurements was quite far from the glass transition zone and closer to the melting region of polysiloxanes [11], where several phenomena may overlap. Imai et al. [33] assigned the increase in tan δ value and the stronger enhancement of the loss modulus to the motion in the interfacial region, which is larger with the increase of nanoparticle concentration. Nevertheless, the agglomerations are also more numerous at a higher amount of nanosilica (Figure 1), reducing the interfacial area. In other works, the increase in the tan δ value in polysiloxane nanocomposites was associated with a decrease in the crosslinking density [34,35]. Indeed, the nanosilica has a high affinity for water, and the water adsorbed on its surface may disturb the synthesis of the polysiloxane during the preparation of nanocomposites. A higher amount of nanosilica will introduce a larger amount of water in the nanocomposites, which may break the siloxane linkages and reduce the crosslinking density [36]. More information on the thermal behavior and the mobility of the polymer chains in function of temperature were obtained from calorimetric and dielectric measurements.

#### 3.2.2. Tensile Test Results

**Figure 5:**
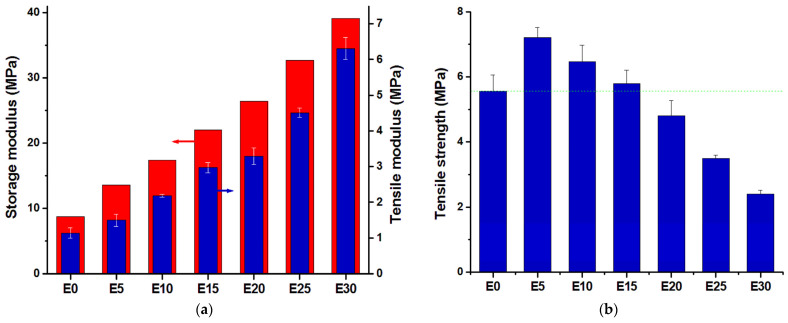
Tensile modulus and storage modulus of nanocomposites at 30 °C (**a**); tensile strength of nanocomposites (**b**).

The results of the dynamical mechanical analysis can be correlated with the mechanical properties determined by tensile tests. The addition of nanosilica led to a strong increase of stiffness, as observed from the increase of the tensile modulus of the nanocomposites (Figure 5a). The increase of the tensile modulus shows the same trend as the storage modulus, highlighting the intense reinforcing effect of nanosilica particles and the immobilization of the polymer chains at the surface of the nanofiller. This is in good agreement with the SEM images showing embedding of nanoparticles in the polymer matrix and no holes at the polymer–nanoparticles interface (Figure 3). Additional proof of the good covering of nanoparticles with polymer may be obtained from water contact angle (CA) measurements. Nanosilica shows a hydrophilic character and its addition to the hydrophobic polysiloxane should decrease the hydrophobic character of its surface. However, a different trend of the CA values was observed, where the contact angle slightly increased with the amount of nanosilica in the nanocomposites, from 100 ± 3.3 for E0 to 104 ± 2.1 for E10, 107 ± 0.36 for E20 and 110 ± 1.3 for E30. This behavior could be explained by the increased roughness [37] of nanocomposites’ surface with the increase of nanosilica content, also evidenced by the increased number and size of nanoparticle agglomerations in the SEM images. Hence, the nanosilica was well covered with the polymer and did not reduce the hydrophobic character of the polymer’s surface.

The tensile strength of the nanocomposites increased only for a nanosilica content up to 15 wt%; above this concentration, the tensile strength of the nanocomposites was lower than that of the reference (Figure 5b). This is a result of the decreased polymer fraction, which can transfer the stress and the important stiffening effect of rigid nanoparticles. In addition, with the increase of nanoparticle concentration, more and larger aggregates will be formed, as demonstrated by SEM images (Figure 1, Figure 2 and Figure 3). They will reduce the polymer–filler interfacial area and interactions, also acting as stress concentrators. All these effects will contribute to a more premature failure and the reduction of tensile strength at higher nanofiller concentrations [12].

**Figure 6:**
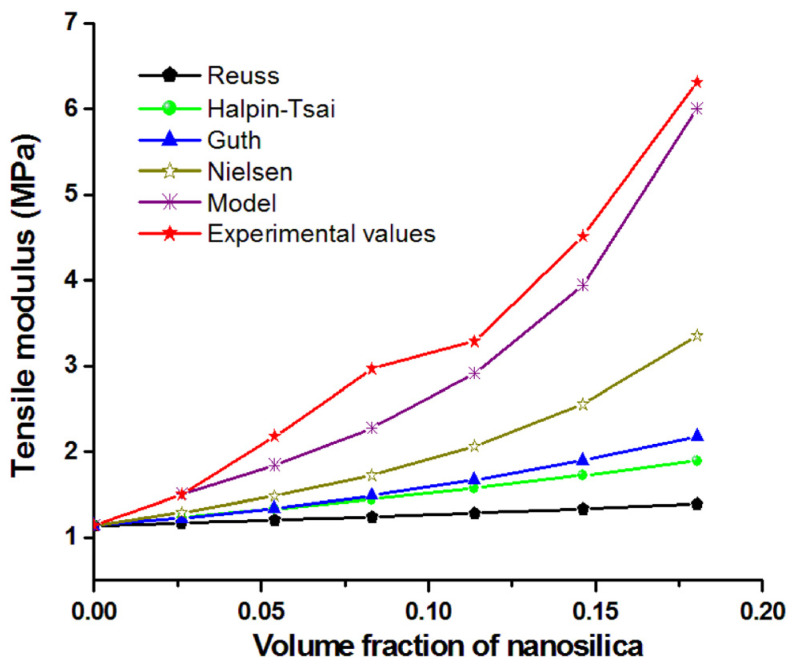
Experimental values of the tensile modulus and theoretical values determined using different models (Reuss, Halpin–Tsai, Guth, Nielsen and Ji).

Several empirical or semi-empirical equations were used for the prediction of the elastic modulus of polymer composites with spherical inorganic particles [38]. Besides the Reuss model (lower limit or series model), several models, such as the Guth model, which considers the interactions between particles in Einstein’s equation [39], the Halpin–Tsai semi-empirical relation [40], the Nielsen equation, which takes into account the formation of aggregates [41], and the Ji model, which considers the effect of the interfacial region [42], were used to predict the variation of the tensile modulus (experimental values) with the concentration of nanosilica particles (Figure 6). The volume fraction of nanosilica was used in all equations. In the Nielsen equation, the aggregation state of nanoparticles, depicted from the SEM images, was used to define Einstein’s coefficient (k) and the maximum packing fraction (φ_m_). Thus, a k value of 4.7 and a φ_m_ value of 0.36, both characteristic to the aggregated particles, were used in the model developed by Nielsen [41,43]. As observed in Figure 6, the modulus determined with the Reuss, Halpin–Tsai or Guth models was quite far from the experimental values, while Nielsen’s equation was closer. One explanation for this is that the models were developed for microparticles and do not consider the effects of interfacial interactions or nanofiller aggregation. Thus, the effect of the introduction of nanoparticle aggregations in the Nielsen model is important; however, it is not enough to predict the tensile modulus.

**Figure 7:**
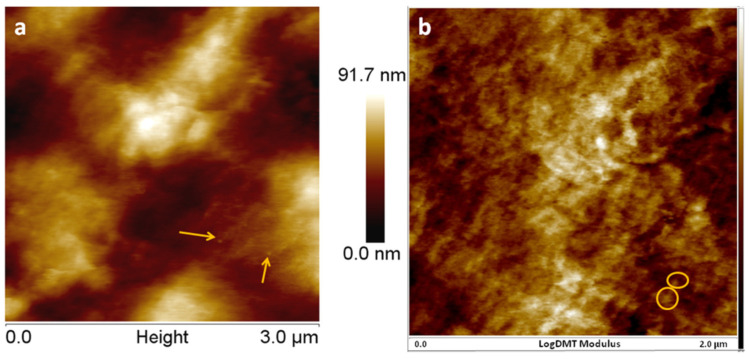
AFM topographic (**a**) and logarithmic modulus (**b**) images of E5.

The three-phase model of Ji [42], which takes into account the effect of the interfacial region in Takayanagi’s two-phase model, led to closer results (Figure 6, Model). Several assumptions were made based on the literature to define the interface thickness and the value of the interface modulus adjacent to the nanoparticles. The interface in nanocomposites is defined as a region in the neighborhood of the nanoparticles’ surface where the properties of the polymer are different compared with that of the matrix [44,45]. The interface thickness in polymer nanocomposites with nanoparticles of 20–50 nm was estimated to be in the range of 10–30 nm by Tanaka et al. [44] and Smith et al. [45], while thicknesses lower than 10 nm were considered in other works [46,47]. For an estimation of the interface thickness closer to that occurring in our nanocomposites, the surface of E5, the nanocomposite with the smallest amount of silica nanoparticles and the least agglomerations, was investigated by atomic force microscopy (AFM) using a MultiMode 8 equipment from Bruker (Madison, WI, USA). The topographic image of E5 (Figure 7a) shows many individual nanoparticles (lighter in color due to their higher density) with a size of about 20 nm (marked with arrows). Interestingly, the AFM modulus (Figure 7a) shows a lighter colored halo around such nanoparticles, which may be interpreted as a region with higher modulus around the nanoparticles compared to the polymer matrix and thus as an interface. Measurements showed that the nanoparticles had a larger diameter by approximately 50% in the AFM modulus image. Therefore, an interface thickness of 10 nm was used in Ji’s model. For the modulus ratio between the interface modulus close to the nanoparticles and that of the matrix, different values between two and five were tested. A three-fold increase of the interface modulus was closer to the experimental values (Figure 6). It should be remarked that Ji’s model, which considers the interfacial region, led to the closest values of the tensile modulus to the experimental ones, highlighting the importance of the interface in these nanocomposites.

### 3.3. Differential Scanning Calorimetry

**Figure 8:**
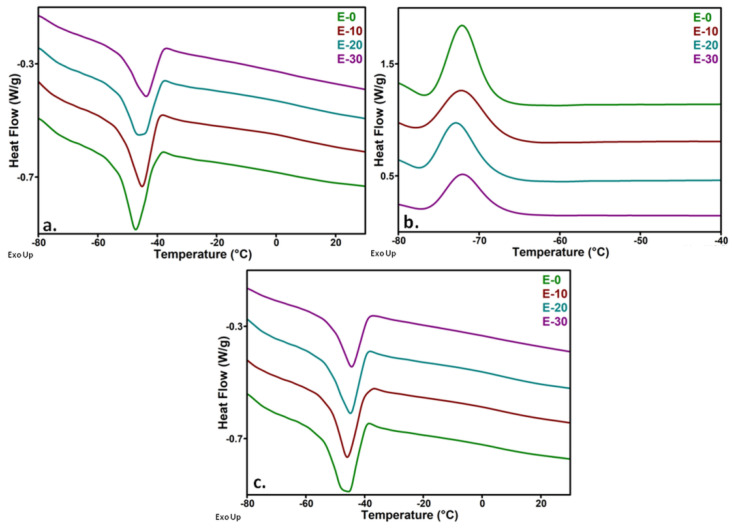
DSC thermograms of nanocomposites from the first heating (**a**), cooling (**b**) and second heating (**c**) cycles.

Thermal events in the temperature range of the DMA measurements were investigated by DSC. The DSC thermograms recorded during the first heating, cooling and second heating for the nanocomposites with 0, 10, 20 and 30 wt% nanosilica are shown in Figure 8.

In this temperature range (−80–50 °C), only one event related to the melting of the crystalline phase (Figure 8a), crystallization (Figure 8b) and remelting (Figure 8c) was noticed. A slight variation of the melting temperature was observed with the increase of nanosilica content in the first heating cycle, and almost no change was observed in the second heating while the crystallization temperature was not modified (Table 2). An increase of crystallinity by up to 6% was noticed in nanocomposites with 10 and 20 wt% nanosilica, and no change was observed for the highest amount of nanofiller (Table 2). It was observed that the melting of the crystalline domains took place between −60 and −40 °C in all the samples. Therefore, the strong decrease of the storage modulus between these temperatures and room temperature is a result of the increased flexibility of the polymer chains released from the ordered crystalline structures.

The increase of the melting temperature by up to 4.0 °C when nanosilica content increased from 0 to 30 wt%, as observed in Table 2, may be due to nanosilica–polysiloxane interactions that restrict the polymer chains’ mobility [48]. However, these differences may also be due to the thermal history of the samples, which is supported by their diminution in the second heating cycle (Table 2, T_m2_).

### 3.4. Thermogravimetric Analysis

**Figure 9:**
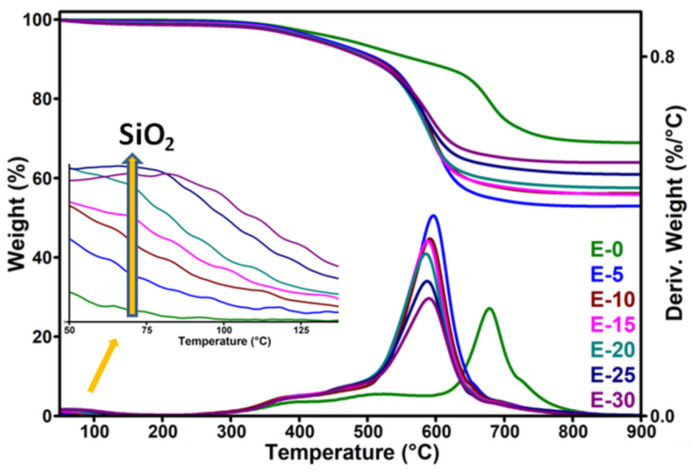
Thermogravimetric and derivative curves of nanocomposites; the detail shows the DTG curves in the range 50–125 °C.

Two major degradation steps were observed in the thermogravimetric and derivative (DTG) curves of the reference E0 (Figure 9). The first step is a low-rate degradation process which appears as a large hump between 300 and 600 °C and is caused by the volatilization of low molecular weight products, mainly oligomers and unreacted monomer [21]. The second step is due to the depolymerization of the polysiloxane by the fracture of Si-O bonds and the formation of cyclic oligomers and crosslinked products [21,49]. The peak temperature corresponding to the main degradation process was 678 °C, similar to other observations [47].

The thermal degradation of polysiloxane in nitrogen atmosphere was accelerated by the addition of nanosilica, as observed from the characteristic parameters listed in Table 3: temperature at 5% weight loss (T_5%_), temperature of maximum degradation rate (T_d_), weight loss at 200 °C (WL_200 °C_) and residue at 1000 °C.

A decrease of T_d_ with 80–90 °C was noticed in the nanocomposites compared to the reference, depending on the nanosilica content. It may be assumed that the water incorporated with the nanosilica particles favored the breaking of the siloxane bonds, decreasing the crosslinking density and the thermal stability [50]. This correlates well with the increase in tan δ values observed by DMA measurements with the increase of nanosilica content (Table 1). However, the decrease of the degradation temperature was not proportional to the amount of nanosilica in the nanocomposites, suggesting contradictory effects. Thus, it may be supposed that this is the effect of the competitive actions of several factors: (i) the silanol groups on the surface of the nanosilica accelerated the thermal degradation, leading to low molecular weight cyclic products [50]; (ii) the nanosilica–polysiloxane interactions improved the dispersion of nanosilica and the thermal stability; (iii) the hydroxyl groups on the nanosilica surface favored the degradation of the siloxane linkage due to the adsorbed water [36]; and (iv) the adsorbed water on the surface of the nanoparticles also favored their agglomeration, leading to a poor dispersion in the polysiloxane and decreased thermal stability [36]. The presence of large nanosilica aggregates in E20 and E30 was demonstrated by SEM, and their presence may disturb the crosslinking process. Similarly, the large number of silanol groups and water may catalyze the breaking of the Si-O bonds, enhancing the decomposition process at higher temperatures. On the contrary, more silanol groups may favor the polymer–nanofiller interactions, leading to improved thermal stability and preventing the release of the volatile degradation products. As a result of opposite factors, similar T_d_ values were observed for the nanocomposites with 10–30 wt% nanosilica. The higher proportion of adsorbed water with the increase of nanosilica concentration in nanocomposites can be better observed from the height of the small peak detected only in the nanocomposites at low temperatures around 100 °C (see the detail in Figure 9).

### 3.5. Dielectric Spectroscopy

**Figure 10:**
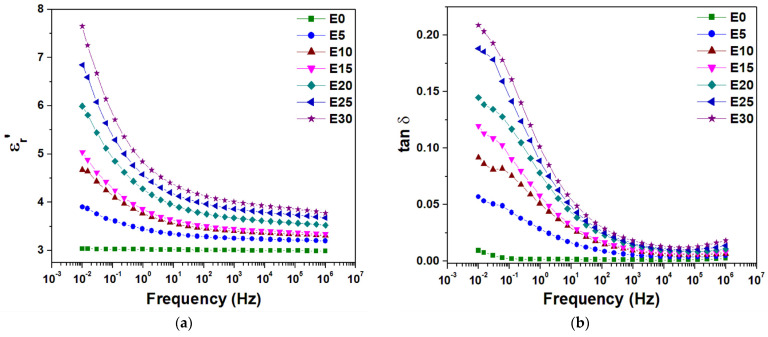
Dependence of ε*_r_’* (**a**) and tan δ (**b**) versus frequency at 30 °C for nanocomposites with different nanosilica content.

Figure 10 shows the frequency variation of ε_r_’ and tan δ determined by dielectric spectroscopy at 30 °C, for the unfilled polysiloxane and the nanocomposites. An important influence of the nanosilica on the dielectric spectrum of the real permittivity ε_r_’ of polysiloxane can be noticed. The, ε_r_’ values increased in the nanocomposites with the increase of nanosilica concentration, mainly due to the new dipoles introduced because of the presence of nanofiller. These new dipoles are mainly localized in the polymer–nanoparticle interface and are due to the adsorbed water in nanocomposites, which increases with the nanosilica concentration as revealed by thermogravimetric analysis. Thus, the adsorbed water facilitated the formation of silanol groups in the interface [21], which act as very mobile side-chain electric dipoles over a broad frequency range of the electric field. Therefore, considering that the large interface of tens of square km per cubic meter of the nanodielectric has the dominant dielectric behavior in this material, the mobile polar groups present in the polymer–nanoparticle interface can lead to an effective real permittivity even higher than both the permittivities of the polymer matrix (≈3) and nanofiller (≈4 [51]).

Another important feature of the spectra shown in Figure 10 is that the increase of ε_r_’ is more important at higher nanofiller concentrations at frequencies lower than 10^3^ Hz. The sharp increase of the real part of the complex permittivity at low frequency can be explained by the superposition of two processes taking place in polysiloxane–nanosilica nanodielectrics, i.e., electrode polarization and translational diffusion of charge carriers. Thus, the large increase of ε_r_’ values accompanied by the presence of a broad loss peak located around 10^−1^ Hz in the case of the nanocomposites emphasizes a space charge accumulation between the electrode and the nanocomposite surface, which is called electrode polarization (EP) [52].The presence of EP increases the real part of the complex permittivity as well as the loss tangent to extremely high values, due to the development of double layers of charge carriers nearby electrodes, which leads to a high electric field in these regions and an extremely low electric field in the bulk of the sample [52,53]. Thus, the high electric field, which appeared in the EP process, leads to the relaxation observed in the low frequency range for nanodielectrics. Besides EP, another typical process that exerts a strong influence on the complex permittivity at low frequencies is ionic conduction due to ion hopping between adjacent potentials. This hopping is long-range at low frequencies below the broad loss peak, whereas at higher frequencies the ions do not have enough time to move, and the hopping is limited to very short-range distances. Such quasi-mobile ions present in polysiloxane–nanosilica nanodielectrics may originate from the adsorbed water, residual catalyst or impurities [35,54].

In addition, the ion transport is accompanied by local motions of different segments of the flexible polymer chains close to the polar nanofiller, and therefore in the interface [16,52,55]. With the increase of nanosilica concentration, the interactions between the nanoparticles and the polymer matrix become stronger, leading to a higher dielectric activity in nanocomposites, e.g., a higher dielectric loss peak at higher nanosilica concentrations, as seen in Figure 10b. Thus, this increased dielectric loss means a higher effort from the electric field in the polarization process; it can be explained by a restriction of the polymer chains’ mobility near nanosilica particles, due to the network of nanoparticles formed in the polysiloxane matrix, determining a leap in stiffness and viscous flow, as revealed by the DMA results. Therefore, the addition of a higher content of nanosilica leads to a supplementary electric field that contributes to the higher dielectric activity seen in nanocomposites. However, it should be noticed that the loss tangent values are lower than 0.02 for all tested nanodielectrics and the unfilled polymer in the radio frequency range between 1 KHz and 1 MHz, whereas an increase of real permittivity by 33% was noticed in E30 compared to E0 at a frequency of 1 KHz.

**Figure 11:**
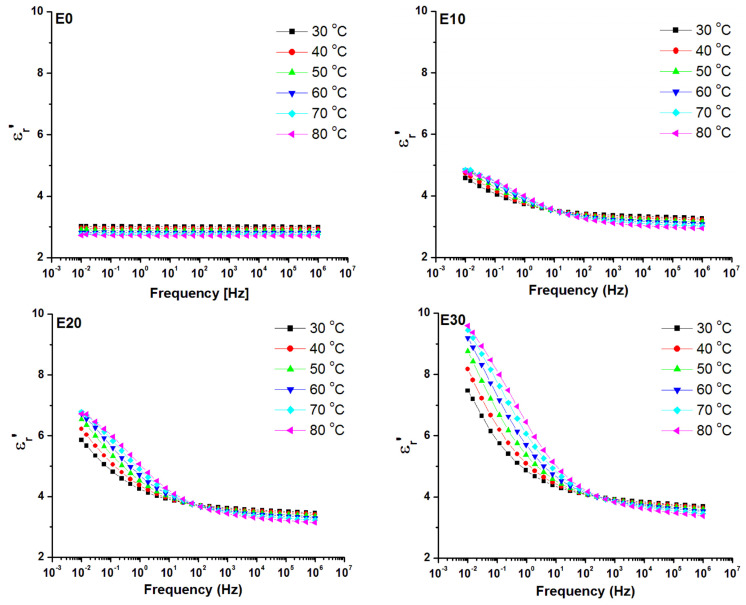
Dependence of ε*_r_’* versus frequency for temperatures in the range 30–80 °C for unmodified polysiloxane (E0) and nanocomposites (E10, E20 and E30).

**Figure 12:**
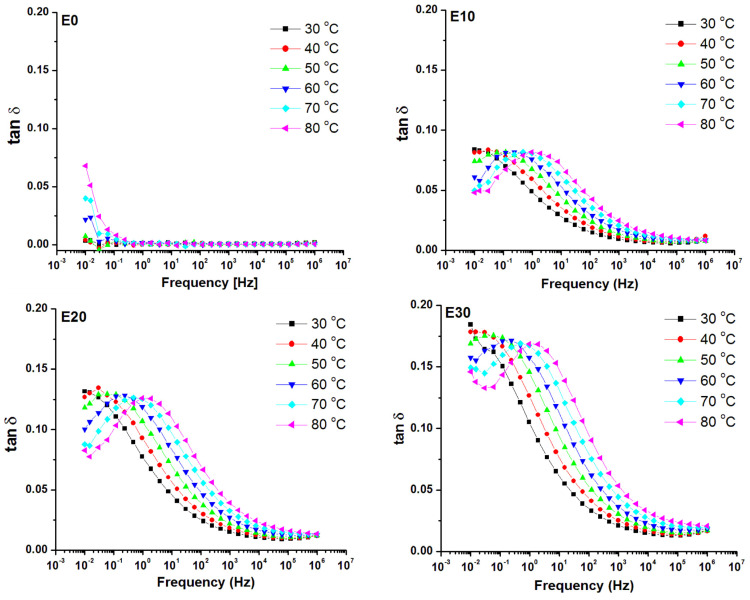
Dependence of tan δ versus frequency for temperatures in the range 30–80 °C for unmodified polysiloxane (E0) and nanocomposites (E10, E20 and E30).

The variation of the temperature may cause thermal activation of some dipolar species and/or ionic conduction, thus affecting the dielectric properties. Therefore, the dielectric behavior of the nanocomposites with the variation in temperature was also analyzed. Figure 11 shows the frequency variation of ε_r_’ with the increase of temperature from 30 °C to 80 °C for E0 (unmodified polysiloxane) and the nanocomposites with 10, 20 and 30 wt% nanosilica. The similar diagrams for tan δ are shown in Figure 12. No variation of permittivity with frequency and a slight decrease with the increase of temperature was noticed for E0, probably due to the thermal agitation [56,57,58]. On the contrary, an increase of ε_r_’ with temperature in the range of low frequencies (up to 10–100 Hz) was noticed in the nanocomposites, the increase being more evident with an increase of nanosilica concentration. However, no significant variation with temperature for the ε_r_’ values at higher frequencies was noticed for the nanocomposites, regardless of the filler concentration.

The dielectric loss (tan δ) variation shown in Figure 12 confirmed that no relaxation process was present in the analyzed frequency range in unmodified polysiloxane. Only a small tan δ variation was observed at low frequencies, due to the influence of DC conduction on the dielectric loss spectrum. A shift of the loss peak, emphasizing the relaxation due to EP, towards higher frequencies with the increase of temperature was observed in the nanocomposites. This shows a thermal activation of the quasi-mobile electric charges, i.e., the hoping of ions assisted by the chain segment motion from the interface. No phase transition occurs between 30 and 80 °C according to the literature [9] and our DSC results (Figure 8). Therefore, the relaxation observed only in the nanocomposites in the range of 10^−1^–10 Hz can be related to the dielectric phenomena in the polymer–nanofiller interface [58]. When the temperature is higher, more ions are activated, raising ε_r_’ more significantly and moving the loss relaxation peak to higher frequencies.

**Figure 13:**
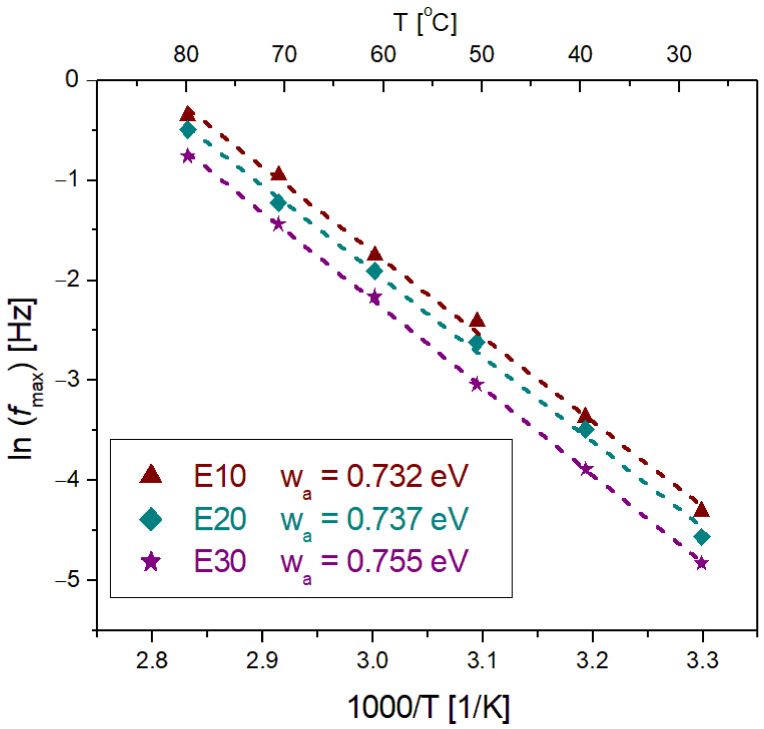
Dependence of log *f_max_* on the inverse temperature for the peak of tan δ (*f*) variation for polysiloxane–nanosilica nanodielectrics with 10, 20 and 30 wt% filler concentrations.

Figure 13 shows the Arrhenius plots displaying the variation of log *f*_max_ with the reciprocal of the temperature (1/*T*) for the relaxation peaks of tan δ seen in Figure 12 for the nanodielectrics with 10, 20 and 30 wt% nanosilica concentration. *f*_max_ denotes the relaxation peak frequency for the relaxation peaks observed in the tan δ frequency variation. The activation energy *w_a_* for the thermally activated relaxation process, determined from these plots for the three nanosilica concentrations, increases slightly with the filler concentration, thus showing an increased dielectric activity as discussed above.

The results shown in Figure 12 indicate that the temperature influence on dielectric losses of polysiloxane–nanosilica nanocomposites is insignificant in the radio frequency (RF) range close to 1 MHz. This, together with the small variation with temperature of ε_r_’ in the RF range, shows a stability of the dielectric properties of these nanocomposites with temperature at radio frequencies, which can lead to a stability in operation of wearable antennas with these nanocomposites as a dielectric substrate. Moreover, the higher ε_r_’ values of the nanocomposites in the RF range, even if the increase with respect to the unfilled polymer is not outstanding, leads to a reduction of antenna dimensions, which is important knowing that wearable devices must be as small as possible [59].

## 4. Conclusions

This is the first study that addresses the improvement of the mechanical and dielectric properties of polysiloxane nanocomposites in a unitary way. New nanodielectrics with a high content of nanofiller were obtained by a simple, environmentally friendly and easily scalable method starting from polysiloxane components and 20 nm nanosilica. The new nanocomposites showed good flexibility and higher stiffness relative to the unfilled polymer, along with increased dielectric permittivity and unmodified dielectric loss at high frequencies. The tensile modulus was well fitted by models that consider the role of the interface. The nanosilica particle–polymer interface influenced the dielectric and mechanical properties of the nanocomposites, showing the importance of interfacial interactions. The new nanomaterials are of interest for wearable electronic devices, due to their mechanical and dielectric properties in the radio frequency range. Further work is needed for a higher increase in the dielectric permittivity considering the improvement of the polymer–nanofiller interface.

## Figures and Tables

**Table 1:** Storage (E’) and loss modulus (E”) of the nanocomposites at different temperatures.

Nanocomposites	E’_−30 °C_, MPa	E’_0 °C_, MPa	E’_30 °C_, MPa	E”_30 °C_, MPa	tan δ_(−__30 °C)_
E0	14.2	10.5	8.7	0.8	0.101
E5	22.3	16.5	13.6	1.1	0.099
E10	31.2	21.7	17.4	1.7	0.111
E15	41.2	28.2	22.0	2.3	0.112
E20	52.5	34.6	26.4	3.1	0.119
E25	75.0	45.8	32.7	4.7	0.134
E30	95.5	56.3	39.1	5.9	0.135

**Table 2:** DSC data for the nanocomposites.

Nanocomposites	First Heating		Cooling		Second Heating		X_c_ (%)
	T_m1_ (°C)	ΔH_m1_ (J/g)	T_c_ (°C)	ΔH_c_ (J/g)	T_m2_ (°C)	ΔH_m2_ (J/g)	
E0	−47.3	17.1	−72.0	29.8	−45.6	17.0	46.2
E10	−45.2	15.8	−71.9	22.8	−46.0	14.8	47.5
E20	−46.1	14.5	−72.7	23.8	−44.9	14.2	49.0
E30	−43.8	11.8	−71.9	17.2	−44.5	11.1	45.6

**Table 3:** Thermogravimetric data of nanocomposites.

Sample	E0	E5	E10	E15	E20	E25	E30
T_5%_, °C	465.0	434.5	423.4	426.9	424.6	419.3	417.2
T_d_, °C	678.2	596.3	591.4	588.0	585.4	587.0	590.0
WL_200 °C_, %	0.2	0.4	0.7	0.8	1.0	1.2	1.3
Residue at 1000 °C, %	68.9	53.1	56.2	55.8	57.4	60.8	64.0

## Data Availability

The data presented in this study are available on request from the corresponding authors.

## References

[1] Liu Y., Pharr M., Salvatore G.A. (2017). Lab-on-Skin: A review of flexible and stretchable electronics for wearable health monitoring. ACS Nano.

[2] Chen J., Liu J., Peng Z., Yao Y., Chen S. (2021). The microscopic mechanism of size effect in silica-particle reinforced silicone rubber composites. Eng. Fract. Mech..

[3] Romasanta L.J., Lopez-Manchado M.A., Verdejo R. (2015). Increasing the performance of dielectric elastomer actuators: A review from the materials perspective. Prog. Polym. Sci..

[4] Yao S., Zhu Y. (2015). Nanomaterial-enabled stretchable conductors: Strategies, materials and devices. Adv. Mater..

[5] Dang Z.-M., Yuan J.-K., Zha J.-W., Zhou T., Li S.-T., Hu G.-H. (2012). Fundamentals, processes and applications of high-permittivity polymer–matrix composites. Prog. Mater. Sci..

[6] Barber P., Balasubramanian S., Anguchamy Y., Gong S., Wibowo A., Gao H., Ploehn H.J., Zur Loye H.-C. (2009). Polymer composite and nanocomposite dielectric materials for pulse power energy storage. Materials.

[7] Dang Z.-M., Yuan J.-K., Yao S.-H., Liao R.-J. (2013). Flexible nanodielectric materials with high permittivity for power energy storage. Adv. Mater..

[8] Ma J., Azhar U., Zong C., Zhang Y., Xu A., Zhai C., Zhang L., Zhang S. (2019). Core-shell structured PVDF@BT nanoparticles for dielectric materials: A novel composite to prove the dependence of dielectric properties on ferroelectric shell. Mater. Des..

[9] Liu Y., Chen J., Jiang X., Jiang P., Huang X. (2020). All-organic cross-linked polysiloxane-aromatic thiourea dielectric films for electrical energy storage application. ACS Appl. Energy Mater..

[10] Shit S.C., Shah P.A. (2013). Review on Silicone Rubber. Natl. Acad. Sci. Lett..

[11] Chiulan I., Panaitescu D.M., Radu E.R., Frone A.N., Gabor R.A., Nicolae C.A., Jinescu G., Tofan V. (2020). and Chinga-Carrasco, G. Comprehensive characterization of silica-modified silicon rubbers. J. Mech. Behav. Biomed. Mater..

[12] Na M., Zhou N.-L. (2014). Synthesis and properties of PDMS/montmorillonite-cetyltrimethylammonium bromide-heparin films. Carbohydr. Polym..

[13] Chen J., Liu J., Yao Y., Chen S. (2020). Effect of microstructural damage on the mechanical properties of silica nanoparticle-reinforced silicone rubber composites. Eng. Fract. Mech..

[14] Faiza, Khattak A., Rehman A.U., Ali A., Mahmood A., Imran K., Ulasyar A., Sheh Zad H., Ullah N., Khan A. (2021). Multi-stressed nano and micro-silica/silicone rubber composites with improved dielectric and high-voltage insulation properties. Polymers.

[15] Kausar A. (2020). Polydimethylsiloxane-based nanocomposite: Present research scenario and emergent future trends. Polym. Plast. Technol. Eng..

[16] Bertasius P., Schaefer S., Macutkevic J., Banys J., Selskis A., Fierro V., Celzard A. (2021). Dielectric properties of polydimethylsiloxane composites filled with SrTiO_3_ nanoparticles. Polym. Compos..

[17] Mathias K.A., Shivashankar H., Shankar B.M., Kulkarni S.M. (2020). Influence of filler on dielectric properties of silicone rubber particulate composite material. Mater. Today: Proc..

[18] Gallone G., Carpi F., De Rossi D., Levita G., Marchetti A. (2007). Dielectric constant enhancement in a silicone elastomer filled with lead magnesium niobate–lead titanate. Mater. Sci. Eng. C.

[19] Manohar Shankar B.S., Hiremath S., Kulkarni S.M. (2019). Influence of conductive and dielectric fillers on the relaxation of solid silicone rubber composites. Mater. Res. Express.

[20] Madidi F., Momen G., Farzaneh M. (2018). Dielectric properties of TiO_2_/silicone rubber micro- and nanocomposites. Adv. Mater. Sci. Eng..

[21] Liu J., Cheng Y., Xu K., An L., Su Y., Li X., Zhang Z. (2018). Effect of nano-silica filler on microstructure and mechanical properties of polydimethylsiloxane-based nanocomposites prepared by “inhibition-grafting” method. Compos. Sci. Technol..

[22] Xue Y., Li X.F., Zhang D.H., Wang H.S., Chen Y., Chen Y.F. (2018). Comparison of ATH and SiO_2_ fillers filled silicone rubber composites for HTV insulators. Compos. Sci. Technol..

[23] Galanti A.V., Sperling L.H. (1970). Morphology and structure of silica agglomerates in silica-reinforced silicone rubber. J. Appl. Polym. Sci..

[24] Yue Y., Zhang H., Zhang Z., Chen Y. (2013). Tensile properties of fumed silica filled polydimethylsiloxane networks. Compos. A Appl. Sci. Manuf..

[25] Mark J.E., Jiang C.-Y., Tang M.-Y. (1984). Simultaneous curing and filling of elastomers. Macromolecules.

[26] Rajan G.S., Sur G.S., Mark J.E., Schaefer D.W., Beaucage G. (2003). Preparation and characterization of some unusually transparent poly(dimethylsiloxane) nanocomposites. J. Polym. Sci. B Polym. Phys..

[27] Yang L., Qiu S., Zhang Y., Xu Y. (2013). Preparation of PDMS/SiO_2_ nanocomposites via ultrasonical modification and miniemulsion polymerization. J. Polym. Res..

[28] Geng C., Zhang Q., Lei W., Yu F., Lu A. (2017). Simultaneously reduced viscosity and enhanced strength of liquid silicone rubber/silica composites by silica surface modification. J. Appl. Polym. Sci..

[29] Liu D., Song L., Song H., Chen J., Tian Q., Chen L., Sun L., Lu A., Huang C., Sun G. (2018). Correlation between mechanical properties and microscopic structures of an optimized silica fraction in silicone rubber. Compos. Sci. Technol..

[30] Liu J., Yao Y., Chen S., Li X., Zhang Z. (2021). A new nanoparticle-reinforced silicone rubber composite integrating high strength and strong adhesion. Compos. A Appl. Sci. Manuf..

[31] Wacker Chemie AG, Solid and Liquid Silicone Rubber Material and Processing Guidelines. http://www.wacker.com/cms/media/publications/downloads/6709_EN.pdf.

[32] Roy N., Bhowmick A.K. (2012). Novel in situ silica/polydimethylsiloxane nanocomposites: Facile one-pot synthesis and characterization. Rubber Chem. Technol..

[33] Imai Y., Inukai Y., Tateyama H. (2003). Properties of Poly(ethylene terephthalate)/layered silicate nanocomposites prepared by two-step polymerization procedure. Polym. J..

[34] Di M., He S., Li R., Yang D. (2006). Resistance to proton radiation of nano-TiO_2_ modified silicone rubber. Nucl. Instrum. Methods Phys. Res. B.

[35] Diao S., Jin K., Yang Z., Lu H., Feng S., Zhang C. (2011). The effect of phenyl modified fumed silica on radiation resistance of silicone rubber. Mater. Chem. Phys..

[36] Lin Y., Wang L., Yin F., Farzaneh M., Liu Y., Gao S. (2019). Comparison of four commonly used high temperature vulcanized silicone rubber formulas for outdoor insulator and their regional adaptability. J. Appl. Polym. Sci..

[37] Chang H., Tu K., Wang X., Liu J. (2015). Fabrication of mechanically durable superhydrophobic wood surfaces using polydimethylsiloxane and silica nanoparticles. RSC Adv..

[38] Fu S.-Y., Feng X.-Q., Lauke B., Mai Y.-W. (2008). Effects of particle size, particle/matrix interface adhesion and particle loading on mechanical properties of particulate–polymer composites. Compos. B. Eng..

[39] Guth E. (1945). Theory of filler reinforcement. J. Appl. Phys..

[40] Halpin J.C. (1969). Stiffness and expansion estimates for oriented short fiber composites. J. Compos. Mat..

[41] Nielsen L.E. (1970). Generalized equation for the elastic moduli of composite materials. J. Appl. Phys..

[42] Ji X.L., Jing J.K., Jiang B.Z. (2002). Tensile modulus of polymer nanocomposites. Polym. Eng. Sci..

[43] Lewis T.B., Nielsen L.E. (1968). Viscosity of dispersed and aggregated suspensions of spheres. Trans. Soc. Rheol..

[44] Tanaka T., Kozaka M., Fuse N., Ohki Y. (2005). Proposal of a multi-core model for polymer nanocomposite dielectrics. IEEE Dielect. El. In..

[45] Smith R.C., Liang C., Landry M., Nelson J.K., Schadler L.S. (2008). The mechanisms leading to the useful electrical properties of polmyer nanodielectrics. IEEE Dielect. El. In..

[46] Sattar M.A. (2021). Interface structure and dynamics in polymer-nanoparticle hybrids: A review on molecular mechanisms underlying the improved interfaces. Chem. Sel..

[47] Siqueira D.F., Schubert D.W., Erb V., Stamm M., Amato J.P. (1995). Interface thickness of the incompatible polymer system PS/PnBMA as measured by neutron reflectometry and ellipsometry. Colloid Polym. Sci..

[48] Gan L., Shang S., Jiang S. (2016). Impact of vinyl concentration of a silicone rubber on the properties of the graphene oxide filled silicone rubber composites. Compos. B Eng..

[49] Chen D., Chen F., Hu X., Zhang H., Yin X., Zhou Y. (2015). Thermal stability, mechanical and optical properties of novel addition cured PDMS composites with nano-silica sol and MQ silicone resin. Compos. Sci. Technol..

[50] Han Y., Zhang J., Shi L., Qi S., Cheng J., Jin R. (2008). Improvement of thermal resistance of polydimethylsiloxanes with polymethylmethoxysiloxane as crosslinker. Polym. Degrad. Stab..

[51] Lin Q., Cohen S., Gignac L., Herbst B., Klaus D., Simonyi E., Hedrick J., Warlaumont J., Lee H., Wu W. (2007). Low dielectric constant nanocomposite thin films based on silica nanoparticle and organic thermosets. J. Polym. Sci. B Polym. Phys..

[52] Tian F., Ohki Y. (2014). Charge transport and electrode polarization in epoxy resin at high temperatures. J. Phys. D Appl. Phys..

[53] Ishai P.B., Talary M.S., Caduff A., Levy E., Feldman Y. (2013). Electrode polarization in dielectric measurements: A review. Meas. Sci. Technol..

[54] Li Y., Tian M., Lei Z., Zhang J. (2018). Effect of nano-silica on dielectric properties and space charge behavior of epoxy resin under temperature gradient. J. Phys. D Appl. Phys..

[55] Lin Y., Wu K., Liu Y., Wang L., Guo M. (2020). Dielectric spectroscopy of aluminium hydroxide particles filled silicone rubber and dielectric model analysis with modified numerical solutions. J. Phys. D Appl. Phys..

[56] Ciuprina F., Plesa I., Notingher P.V., Rain P., Zaharescu T., Panaitescu D. Dielectric properties of LDPE-SiO2 Nanocomposites. Proceedings of the 10th IEEE International Conference on Solid Dielectrics.

[57] Ciuprina F., Andrei L. Water and heat exposure influence on dielectric response of LDPE-Al_2_O_3_ nanocomposites. Proceedings of the International Symposium on Fundamentals of Electrical Engineering (ISFEE).

[58] Andrei L., Ciuprina F., Radu E., Gabor A., Panaitescu D. Dielectric performances of LSR-SiO_2_ nanocomposites for wearable antennas substrate. Proceedings of the 7th International Symposium on Electrical and Electronics Engineering (ISEEE).

[59] Paracha K.N., Rahim S.K.A., Soh P.J., Khalily M. (2019). Wearable antennas: A review of materials, structures, and innovative features for autonomous communication and sensing. IEEE Access.

